# Evaluation of patients with oral squamous cell carcinoma treated by radical irradiation with mold radiotherapy using a customized device: a retrospective clinical study

**DOI:** 10.1186/s13256-022-03311-z

**Published:** 2022-04-29

**Authors:** Tadahide Noguchi, Yasushi Sugiura, Akihiro Dohi, Manabu Yamada, Naruo Okada, Ken-ichi Sasaguri, Satoru Takahashi, Yoshiyuki Mori

**Affiliations:** 1 Department of Dentistry, Oral and Maxillofacial Surgery, Jichi Medical University, 3311-1 Yakushiji , Shimotsuke-shi, Tochigi 329-0498 Japan; 2 Department of Dentistry, Oral and Maxillofacial Surgery, National Hospital Tochigi Hospital, 1-10-37 Nakatomatsuri, Utsunomia, Tochigi 320-8580 Japan; 3 Department of Radiology, Jichi Medical University, 3311-1, Yakushiji, Shimotsuke-shi, Tochigi 329-0498 Japan

**Keywords:** Mold therapy, Remote after loading system, Oral cancer, Customized device, Radical irradiation

## Abstract

**Background:**

It is very important to determine the indication of mold radiotherapy for the radical treatment of oral cancer. We investigated eight patients with oral squamous cell carcinoma who were treated by radical irradiation with mold radiotherapy using a customized device.

**Methods:**

The subject is a case of curable superficial oral cancer of a few millimeters, or cancer of a size that can be cured by the placement of the radiation source. Of the eight patients, six were male and two were female, aged 64–93 years (mean, 78.9 years; median, 73.5 years). The primary sites were the buccal mucosa in three cases, gingiva in two cases, and floor of the mouth, soft palate, and lower lip in one case each. Five cases were in the T1 stage, and the remaining three cases were in T2. With respect to thickness, seven cases were of the superficial type and could not be detected by magnetic resonance imaging or computed tomography, and the remaining case showed a tumor thickness of 7.5 mm. All cases were diagnosed as squamous cell carcinoma by biopsy. Radical irradiation using mold radiotherapy was planned for all eight patients. Irradiation was delivered in 9–10 sessions, with a total dose of 45–50 Gy.

**Results:**

Complete response was attained in six of the eight patients and partial response was observed in two patients, requiring additional treatment.

**Conclusion:**

Since all patients with complete response had superficial cancers, we hypothesized that superficial cancers of the oral cavity with thicknesses of few millimeters could be indicated for mold irradiation. This method can be applied in complicated cases and older patients in whom surgery or chemotherapy may not be feasible. We believe that the results of our clinical studies will be of great help in choosing this method.

## Background

The choice of treatment for oral cancer by surgery is not just governed by factors such as the risk of complications and age. The oral cavity performs several important functions, such as speech, mastication, and swallowing, and radiotherapy may need to be selected considering the esthetic aspects.

Mold radiotherapy is delivered from a source close to the tumor, thereby limiting the area of irradiation and allowing the treatment to be completed in a short period. This method of treatment can be employed in complicated cases and older patients in whom the feasibility rate of surgery and/or chemotherapy may be limited.

Previously, we reported a method of mold irradiation using a customized device and specific methods for varied treatment modalities, such as radical irradiation, adjuvant irradiation before and after surgery, and palliative irradiation [1].

In this study, we investigate eight cases of oral squamous cell carcinoma treated by radical irradiation using mold radiotherapy and report the indications and limitations of radical irradiation with mold radiotherapy.

## Methods

From October 2006 to December 2019, we diagnosed oral squamous cell carcinoma and performed mold radiotherapy using a customized device in 18 cases. Herein, we report eight cases that were treated by radical irradiation using mold radiotherapy (Table 1).

**Table 1:** Summary of the eight patients (No. 1)

Case	Sex	Age	Primary sites	Incipient/recurrence	TNM classification	Medical history	History of smoking	History of drinking	Physical condition	Neurology examination	Employment history
1	M	78	Buccal mucosa	Recurrence (postirradiation)	rT1N0M0	Renal cancer Bladder cancer	Ever smoker	None	Normal	Normal	Farmer
2	M	93	Floor of mouth	Recurrence (postirradiation)	rT1N0M0	Atrial fibrillation Cerebral infarction	Ever smoker	Ever drinker	Wheelchair use	Night delirium	Company employee
3	M	74	Buccal mucosa	Incipient	T1N0M0	Colon cancer Lacrimal cancer Renal failure	Ever smoker	Ever drinker	Normal	Normal	Company employee
4	M	70	Soft palate	Incipient	T2N0M0	Aspiration pneumonia Cerebral hemorrhage	Ever smoker	None	Wheelchair use	Hemiplegia	Company employee
5	F	73	Lower lip	Incipient	T2N0M0	Colon polyp	None	None	Normal	Normal	Housewife
6	F	71	Upper gingiva	Recurrence (post-chemoradiation)	rT1N0M0	Ovarian cyst	None	None	Normal	Normal	Company employee
7	M	88	Buccal mucosa	Recurrence (postirradiation)	rT2N0M0	Myocardial infarction COPD Lung cancer	Smoker	None	Wheelchair use	Normal	Unemployed
8	M	64	Upper gingiva	Incipient	T1N0M0	Gastric cancer Esophageal cancer	Ever smoker	Ever drinker	Normal	Normal	Company employee

*COPD* chronic obstructive pulmonary disease

The eight patients comprised six male and two female patients, aged 64–93 years (mean, 78.9 years; median, 73.5 years).

The primary sites of oral squamous cell carcinoma were the buccal mucosa in three cases, gingiva in two cases, and floor of the mouth, soft palate, and lower lip in one case each.

Of the eight cases, four were incipient cases, three were recurrence cases after radiotherapy, and one was a recurrence case after chemoradiotherapy.

Regarding the “T” element of the TMN classification, five cases were in the T1 stage, and the remaining three cases were in T2. With respect to thickness, seven cases were of the superficial type and could not be detected by magnetic resonance imaging (MRI) or computed tomography (CT), and the remaining case showed a tumor thickness of 7.5 mm.

Esthetics was a major concern in the case of labial carcinoma; surgery was refused by a patient with maxillary gingival carcinoma, and six cases were identified as difficult to operate due to potential complications and age. Hence, radical irradiation using mold radiotherapy was planned for all eight patients. Table 1 presents the smoking/drinking history, physical condition and neurological examination, and employment history of the patients. Laboratory finding are presented in Table 2.

**Table 2:** Laboratory findings of the eight patients

Case	Leukocytes (×10^3^ μL)	Erythrocytes (×10^4^ μL)	Hemoglobin (g/dL)	Platelets (×10^4^μL)	BUN (mg/dL)	Creatinine (mg/dL)	ALT (U/L)	AST (U/L)
1	3.6	382	8.5	31.3	20	1.01	14	6
2	4.3	482	13.1	10.9	25	1.29	21	7
3	7.0	325	10.8	19.7	60	3.31	21	13
4	9.2	401	13.1	21.9	17	0.65	52	122
5	3.0	418	13.4	10.3	15	0.60	19	19
6	3.6	383	12.0	23.4	15	0.48	25	24
7	2.6	340	11.4	14.0	10	0.69	18	11
8	5.1	421	12.5	21.6	13	1.05	49	72

If the tumor was located in a region where impression-making was possible, a plaster working model was prepared by recording an impression of the tumor and associated areas. A plastic plate was fabricated using 1.5-mm Erkodur (Erkodent Inc., Germany). The range of irradiation was set just above the marked tumor, and catheters (1.7 mm in diameter and 200 mm in length) were fixed with resin at parallel intervals of 10 mm for delivering the source.

The plastic plate was used as a fixation device to ensure reproducibility and was supported by the teeth in dentate patients.

In cases of edentulous patients and patients with few remaining teeth, the device was stabilized by occluding with a similar plate fabricated on the opposite jaw. In cases of carcinoma of the buccal mucosa, a 2-mm thick acrylic plate was fixed to the plastic plate, and the combined plate was placed in the correct orientation in the oral cavity. Thereafter, the tumor area was marked, and the catheters were fixed.

Irradiation was performed with a remote after-loading system (RALS; Multisource, Eckert & Zieglen, BEBIG Corporation, Germany), by which ^60^Co is remotely delivered to the affected area. The required doses of brachytherapy were delivered by HDR plus (Eckert & Zieglen, BEBIG Corporation, Germany). Dose assessment points were essentially set at a depth of 5 mm from the surface of the tumor. When a fixture is involved, the distance from the radiation source is often 6–7 mm due to the added thickness of the device.

Irradiation was delivered at a dose of 5 Gy twice daily for a total dose of 45–50 Gy in 4.5–5 days (in this case, the biologically effective dose was calculated as *α*/*β* = 10 Gy, which was equivalent to approximately 56–64 Gy in a standard 2 Gy per fraction equivalent dose, according to the linear-quadratic model).

In addition, surgical gauze was interposed as a spacer between the device and oral mucosa to minimize the exposure of the oral mucosa other than the affected area.

The oral examination after mold radiotherapy was mainly a visual inspection, and CT and MRI examinations were performed regularly for the detection of recurrence.

## Results

Table 3 presents the treatment results for all eight patients. The treatment was completed in all eight patients. Complete response (CR) was observed in six of the patients (CR rate 75%), all of which were superficial cancers and could not be detected by magnetic resonance imaging (MRI) or computed tomography (CT). Of the two cases of partial response, one case with lower lip cancer had a thickness of 7.5 mm. This case showed CR after an additional session of mold radiotherapy by direct intratumor puncture of the catheter, and the other case was surgically treated by partial resection of the residual tumor with no evidence of recurrence until now. Acute mucosal reactions in the irradiated field were observed in all cases, and Grade 2 mucositis peaked at 1–2 weeks after irradiation and continued until approximately 5 weeks. All cases were treated by azulene gargles and regular oral care. All patients continued oral intake satisfactorily. No local or systemic short/long term side effects, including osteoradionecrosis of the jaws, were observed during the follow-up period.

**Table 3:** Summary of the eight patients (No. 2)

Case	Size (mm)	Thickness	Number of catheters	Dose rate (Gy/fr/days)	Systemic side effects Short term/long term	Local side effects Short term/long term	Response	Follow-up (months)
1	20 × 10	Superficial^a^	3	50/10/5	None/none	Mucositis/none	CR	138
2	9 × 5	Superficial^a^	2	50/10/5	None/none	Mucositis/none	CR	94
3	19 × 14	Superficial^a^	2	50/10/5	None/None	Mucositis/none	CR	35
4	22 × 21	Superficial^a^	3	50/10/5	None/none	Mucositis/none	CR	19
5	13 × 12	7.5 mm	2	50/10/5	None/none	Mucositis/none	PR	19
6	5 × 5	Superficial^a^	2	45/ 9/4.5	None/none	Mucositis/none	CR	12
7	21 × 5	Superficial^a^	3	50/10/5	None/none	Mucositis/none	PR	69
8	10 × 8	Superficial^a^	3	50/10/5	None/none	Mucositis/none	CR	84

^a^Difficult to measure on images

*CR* complete response,* PR* partial response

## Case presentation (Case 4)

**Fig. 1:**
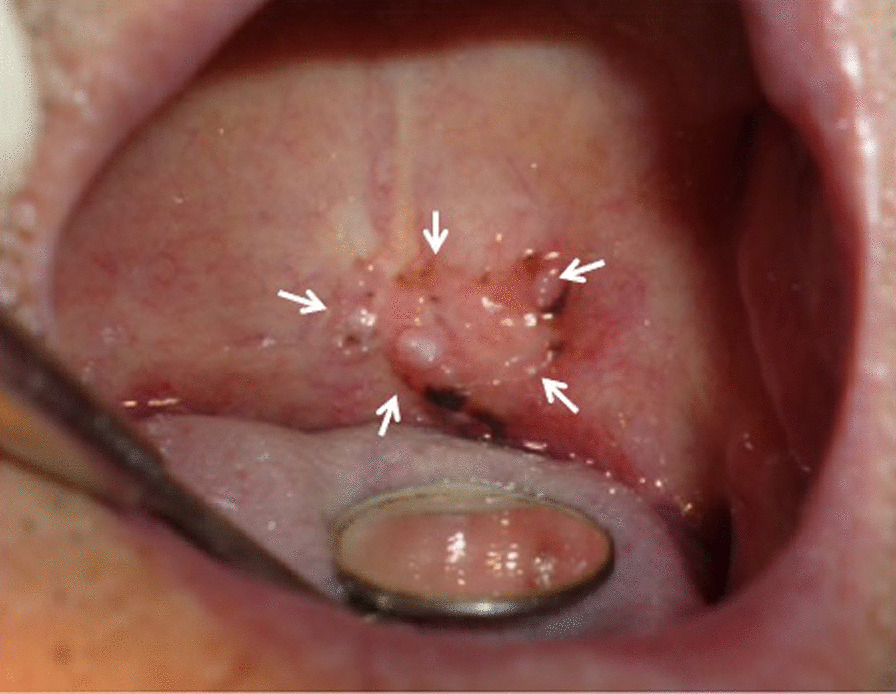
Tumor in the middle of the soft palate (white arrows)

A 70-year-old man was referred to our hospital because of mass lesions with erosion in the center of the soft palate (Fig. 1). Biopsy revealed well-differentiated squamous cell carcinoma. The patient experienced repeated episodes of aspiration pneumonia due to hemiplegia caused by a cerebral infarct. Hence, surgery under general anesthesia was determined difficult to perform.

**Fig. 2:**
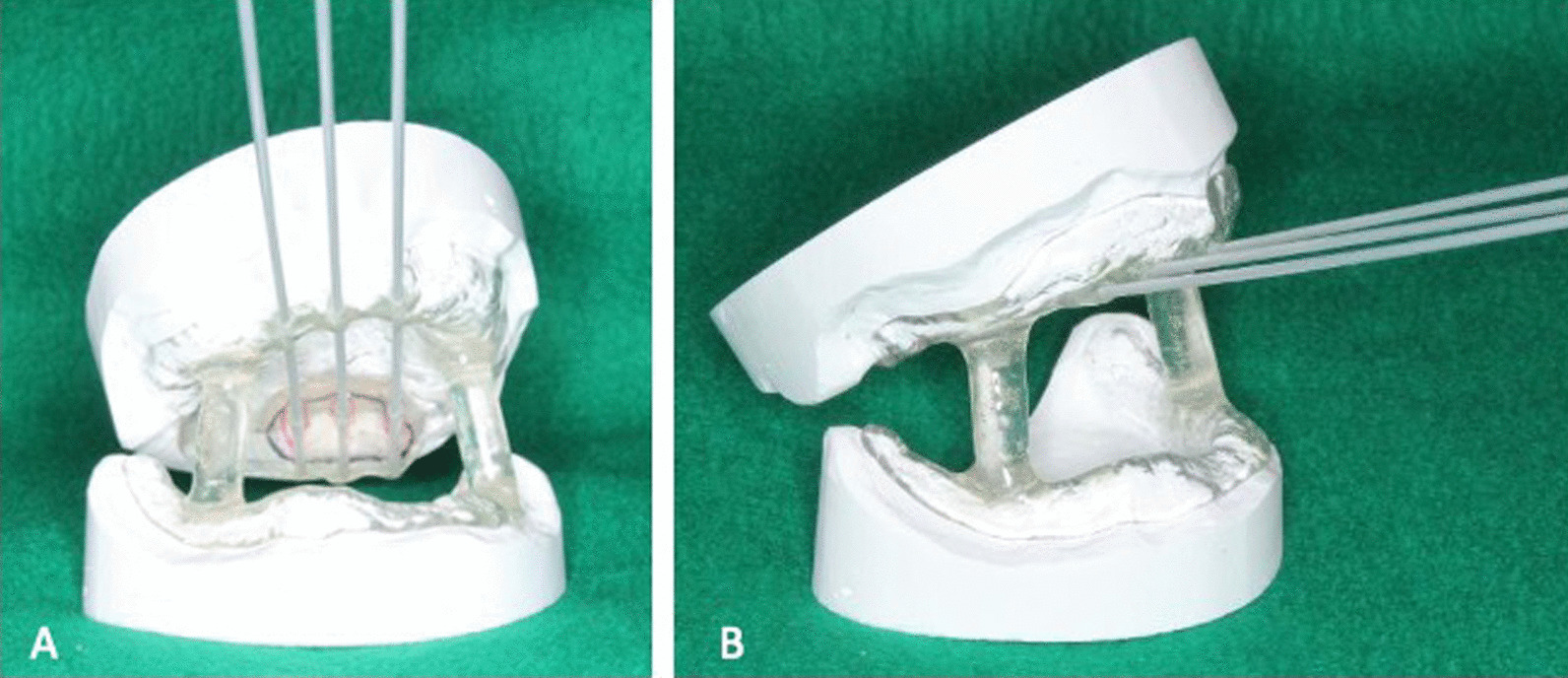
Tumor boundary marked on the surface of the device. Three catheters are embedded in parallel (**A**). The device is stabilized by occluding with a similar device fabricated for the mandible (**B**)

The patient was treated with radical irradiation using mold radiotherapy with a customized device. Three catheters were embedded in the plastic plate, which was fabricated from acrylic resin. The locations of the catheters were determined according to the tumor boundaries seen on the plaster model (Fig. 2).

**Fig. 3:**
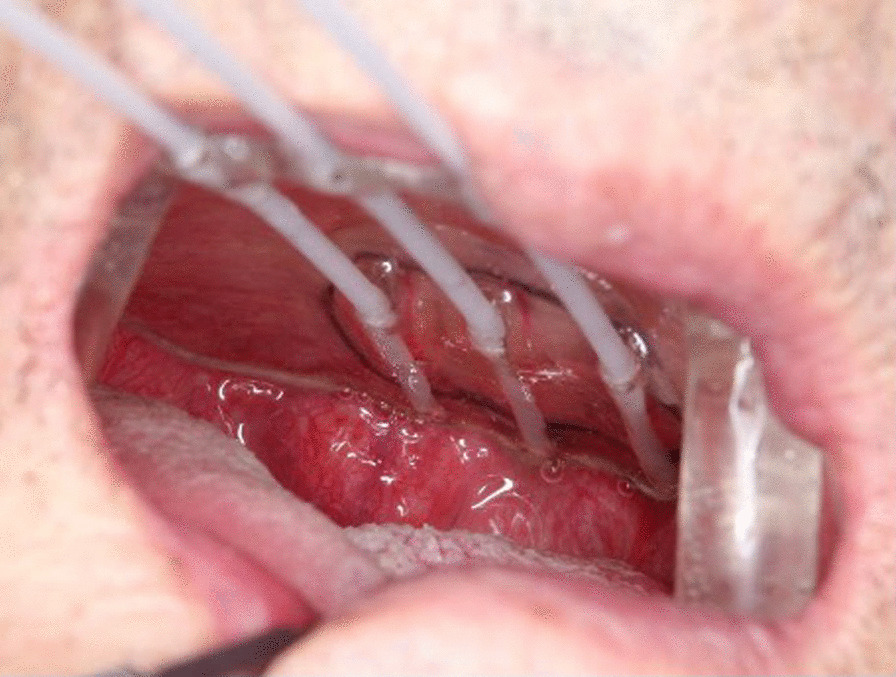
Intraoral placement of the device

We bent the catheters slightly, ensuring that the radiation source was not obstructed. The device was placed in the oral cavity and mold radiotherapy was performed with Multisource (Fig. 3). A total dose of 50 Gy was administered in 5 days. Radiation mucositis peaked at 10 days after treatment. After the treatment, aspiration pneumonia did not recur, and no eating disorders were observed.

**Fig. 4:**
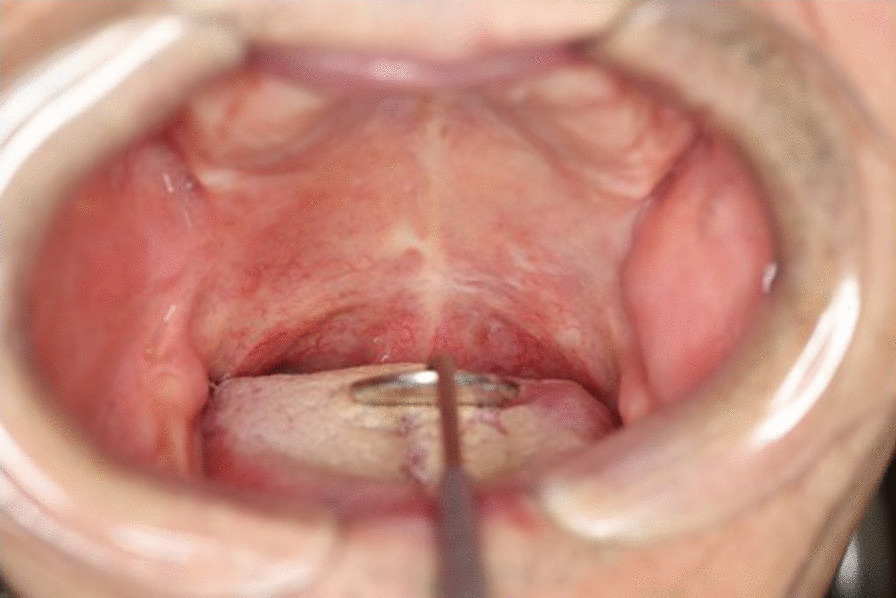
Intraoral findings 19 months after treatment. There is no sign of tumor recurrence

There was no sign of tumor recurrence during the 19 months of follow-up (Fig 4).

## Discussion

Mold radiotherapy is high-dose-rate brachytherapy using a RALS that delivers high doses of iridium or cobalt from an applicator remotely.

The most common indications for RALS in the oral cavity are tongue and soft tissue lesions including the lips and floor of the mouth, for which plastic catheters can be used for puncture [2–6]. However, catheter insertion is difficult for palatal and gingival lesions and, therefore, not indicated in such cases.

Recently, RALS is being used with customized mold radiotherapy that does not require puncture for oral tumors. Several cases of oral cancers have been treated with mold radiotherapy using a customized device [7–12]. Therefore, we fabricated a customized device that can fix the plastic catheters used for delivering the radiation source in a highly reproducible position for the use of the RALS in narrow and complicated anatomical regions, such as the oral cavity.

The most important factors determining the indications of mold radiotherapy are the primary site and thickness of the tumor. Regarding the primary site of the tumor, mold radiotherapy is indicated in regions where the device can be easily placed and in cases where the plastic catheter can be easily fixed without significant anatomical flexion. Therefore, it is indicated in gingival and palatal lesions, especially hard palatal lesions, but other sites have also been reported to be treated using mold radiotherapy [6–11].

The customized device is a plastic plate, which is fabricated on the working plaster model of the tumor, and the catheter is placed just above the tumor. The catheter should be attached without any bends to prevent the obstruction of the radiation source. Irradiation at a reproducible location is possible by fixing the plastic plate to the teeth in patients with an adequate number of remaining teeth. In patients with edentulous jaws or few remaining teeth, an additional plastic plate is fabricated for the opposite arch, and occlusion is simulated for ensuring the stability of the plate.

In addition, irradiation at a reproducible location for soft tissue lesions, such as those of the tongue, floor of the mouth, and buccal mucosa, can be ensured by using plastic plates with adhered acrylic plates and placing the plastic catheter under pressure from the acrylic plate.

Regarding the thickness of the tumor as a factor in determining the indications of radical irradiation using mold radiotherapy, this type of treatment is indicated in superficial cancers with a thickness of a few millimeters. As six of our eight cases showed CR and all six CR cases were superficial cancers, the assessment points were essentially set at a depth of 5 mm from the surface of tumor. These cases of superficial cancer could not be detected by CT or MRI.

Nishimura *et al*. [9] reported that because the isodose at 10 mm from the surface of the mold is approximately half of the reference dose at 5 mm, the radiation dose to the residual tumor cells at sites further from the tumor surface might be insufficient. Mold radiotherapy is indicated in early-stage superficial cancers and not in tumors thicker than 5 mm.

In recent years, the ultrasound probe has become smaller, and ultrasound imaging of the oral cavity is now possible.

For superficial oral cancers, it is indispensable to measure the thickness of the tumor by ultrasound and evaluate whether mold radiotherapy can be applied.

In cases with thicker lesions, it is necessary to consider alternate treatment methods that provide enhanced therapeutic effects, such as combination with external irradiation or low-dose rate.

Of the two cases of recurrence, the case with lower lip cancer had a thickness of more than 5 mm; hence, irradiation was performed with the catheter sandwiched between two sides of the tumor. However, CR was not attained. The lower lip is a movable structure, and there is no method of adequate fixation, suggesting that it could not be fixed in a reproducible position. Therefore, the irradiation dose might not have been sufficient.

The other was a recurrence case after external irradiation of buccal mucosal cancer, and the boundaries of the tumor were unclear. Hence, sufficient dose could not be delivered. In cases of recurrence after external irradiation, accurate determination of the tumor boundaries is difficult due to scarring, erosion, and dilation of capillaries associated with the mucous membrane. Such lesions with unclear boundaries show high risk of recurrence. Such cases should be dealt with by using alternate methods, such as wider setting of the irradiation range.

Mold radiotherapy can be applied to patients with complications, and older patients in whom surgery or chemotherapy may not be possible, with consideration of other existing factors.

Cases with tumor thickness exceeding 5 mm, unclear boundaries, difficult to determine irradiation ranges, anatomical limitations to catheter placement, and nonreproducible catheter positions are not indicated for mold treatment.

## Conclusions

To summarize, we reported eight cases of oral squamous cell carcinoma treated by radical irradiation using mold radiotherapy with a customized device. Mold radiotherapy using a customized device can be applied in complicated cases and older patients in whom surgery or chemotherapy may not be feasible, especially for superficial oral cancers with a thickness of a few millimeters and with clear boundaries.

## Data Availability

The data are not available for public access because of patient privacy concerns, but are available from the corresponding author on reasonable request.

## Abbreviations

MRI: Magnetic resonance imaging
CT: Computed tomography
CR: Complete response
